# Emerging Psychotropic Drug for the Treatment of Trigeminal Pain: Salvinorin A

**DOI:** 10.3390/ph17121619

**Published:** 2024-11-30

**Authors:** Geovanna Nallely Quiñonez-Bastidas, Lucia Elhy Grijalva-Contreras, Selene Isabel Patiño-Camacho, Andrés Navarrete

**Affiliations:** 1 Centro de Investigación y Docencia en Ciencias de la Salud, Universidad Autónoma de Sinaloa, Eustaquio Buelna 91, Burócrata, Culiacan 80030, Mexico; 2 Programa de Licenciatura en Fisioterapia, Universidad Estatal de Sonora, Unidad Académica Hermosillo, Hermosillo 83100, Mexico; lucia.grijalva@ues.mx; 3 Facultad de Ciencias Químico-Biológicas, Universidad Autónoma de Sinaloa, Ciudad Universitaria, Culiacan 80013, Mexico; selene_patino@uas.edu.mx; 4 Departamento de Farmacia, Facultad de Química, Universidad Nacional Autónoma de México, Ciudad Universitaria, Coyoacán, Ciudad de Mexico 04510, Mexico; anavarrt@unam.mx

**Keywords:** trigeminal neuralgia, salvinorin A, κ-opioid receptor agonist, *Salvia divinorum*

## Abstract

Trigeminal neuralgia (TN) is chronic pain caused by damage to the somatosensorial system on the trigeminal nerve or its branches, which involves peripheral and central dysfunction pain pathways. Trigeminal pain triggers disruptive pain in regions of the face, including within and around the mouth. Besides clinical experiences, translating the language of suffering into scientific terminology presents substantial challenges. Due to the complex and multifactorial pathophysiology underlying trigeminal pain, elucidating its social impact presents significant difficulties. Carbamazepine and oxcarbazepine are first-line treatments for TN, achieving approximately 50% pain reduction in 60–70% of treated patients. However, their efficacy is often limited by common side effects, such as dizziness, vertigo, nausea, seizures, and cognitive symptoms. In some cases, patients experience severe side effects, including myelosuppression, hyponatremia, hormonal imbalances, liver toxicity, suicidal ideation, teratogenicity, and other adverse reactions. Given these clinical limitations, the search for new painkiller candidates continues. Hence, we focused this review on salvinorin A (SalA), a natural agonist of κ-opioid receptors (KORs), which demonstrated anti-nociceptive, anti-inflammatory, and anti-neuropathic properties in various experimental models of the spinal sensory system. Furthermore, preclinical evidence indicates that SalA does not induce dependence and demonstrates a favorable toxicological and safety profile in comparison with currently marketed opioid drugs. We propose Salvinorin A as a promising candidate for treating trigeminal neuralgia, offering the potential for reduced adverse effects.

## 1. Introduction

According to the International Headache Society, trigeminal neuralgia (TN) is a disorder characterized by recurrent unilateral brief electric shock-like pain, abrupt in onset and termination, limited to the distribution of one or more divisions of the trigeminal nerve, and triggered by innocuous stimuli [1]. 

The trigeminal nerve is the largest cranial nerve, and its branches provide the main sensory nerves of the anterior two-thirds of the head and face [2]. TN can be classified according to classical etiology when it involves the vascular compression of the trigeminal nerve root, secondary when TN is an underlying neurological disease, or idiopathic when pain occurs without an apparent cause. In all cases, TN is diagnosed by unilateral and pain paroxysm on trigeminal nerve branches, followed by continuous pain in a few cases. The image and neurological tests might be applied to determine the etiology and establish the neuropathic condition [3]. TN is commonly characterized by severe pain, described as sharp, electric, and shock-like sensations, with paroxysmal episodes ranging from a few seconds to no more than 2 min [4]. Sensory abnormalities during neurological examinations are present in almost 29% of cases, such as hyperesthesia, allodynia, and hyperalgesia, whereas patients also report other symptoms, such as numbness, prickling, and other paresthesia, and at least 31% of patients report autonomic symptoms [5]. Due to paroxysmic symptoms, TN is related to the disability index in up to 45% of patients who suffer from this disorder. Parallelly, it is associated with comorbidities such as anxiety and depression in 37.5% and 50%, respectively, as well as catastrophic or damaging ideas of pain in 78% of cases [6]. The coexistence of TN and mood disorders hallmark the quality of life of patients. Despite the prevalence of TN being low, at 0.03 to 0.3% [7], patients can suffer from 10 to 50 attacks of pain per day [5,8]. Moreover, epidemiological studies indicated a strikingly higher prevalence in women compared with men (3:1 proportion), suggesting a modulator role of gonadal hormones [9]. Another epidemiological characteristic is that 90% percent of cases initiate after 40 years old [7]. 

According to reports, not all cases have efficient TN management [6]. However, according to the guidelines on TN management, 90% of patients respond to sodium channel blockers, carbamazepine, and oxcarbazepine, which are the first-line options for pharmacological treatment [10]. Research indicates that 94% of patients who discontinued treatment for TN did so due to adverse side effects, emphasizing the urgent need for new drugs with improved tolerability and a reduced side-effect profile [5,11]. Accordingly, extensive efforts have been made to introduce innovative pharmacological approaches for treating trigeminal pain.

κ-opioid receptor (KOR) agonists are known to induce analgesia, which is not a new finding. However, the opioid crisis has positioned the KOR receptor as an emerging target for pain management, with the potential to reduce the side effects and lethal risks associated with conventional drugs that act on µ-opioid receptors (MORs) [12,13,14]. In this regard, Salvinorin A (SalA) is a potent KOR agonist, demonstrating its therapeutic potential to treat pain in several experimental models [15,16,17]. In this review, we provide an update on the pharmacokinetic (PK), pharmacodynamic (PD), and toxicology characteristics of SalA; its antinociceptive and anti-neuropathic properties on several experimental pain models; as well as the synthetic analogs with therapeutic antipain use; followed by the physiopathology of TN, TN-induced models, and the cross-talk between SalA and trigeminal pain regulation. Moreover, we combined all preclinical evidence between trigeminal pain regulation and the mechanism of action that underlies the anti-pain properties of SalA to finally focus on the exploration of the possible potential of this compound for treating TN.

## 2. Salvinorin A, a Compound Isolated from Ethnobotanical Medicine

**Figure 1:**
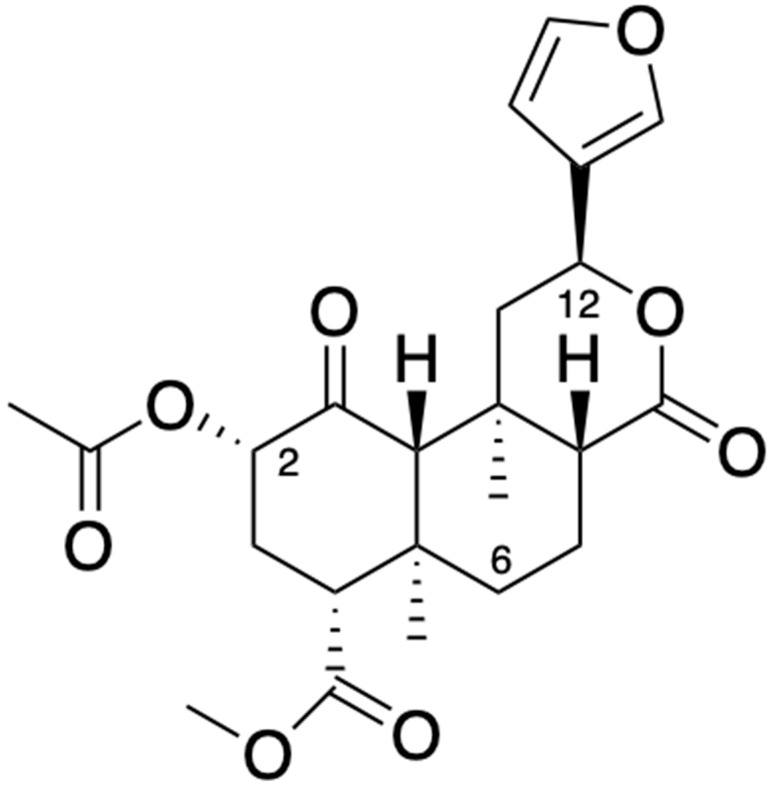
Chemical structure of Salvinorin A. Created in ChemDraw Professional 16.0.

SalA is extracted from the leaves of *Salvia divinorum*, an endemic herb from the Mazatec region in Oaxaca, Mexico, that also grows in South America. This plant was used by healers or people from that region with empirical knowledge of ethnobotanical medicine to treat anemia, headache, rheumatism, diarrhea, and the “semi-magical” disease panzón de Borrego. Also, Mazatec shamans introduced the plant for divine rituals, which was the most common use [18]. SalA is a non-nitrogenous diterpenoid compound (Figure 1) that belongs to the salvinorins group B–J found in the plant [19,20,21,22,23,24]. Interestingly, SalA is the primary compound found in the plant leaves. Samples from the Mazatec region show a variation range between 0.89 and 7.6 mg of SalA per gram of dried leaves [25,26]. Several techniques were described for the extraction of SalA. Dried leaves of *Salvia divinorum* were extracted with ether using a Soxhlet apparatus. When the extract was partitioned between hexane and aqueous methanol, a fraction of almost 10% SalA was obtained by dried weight [27]. In another method, 250 g of dried *Salvia divinorum* leaves were pulverized and then macerated with acetone for 4 h. After filtration, the residue was washed with 3 × 50 mL of 1:3 isopropanol–hexane. The remaining solid was dissolved in dichloromethane and dry-loaded onto silica gel. Flash chromatography using a gradient from hexanes to 1:3 hexanes–ethyl acetate afforded fractions of semi-pure SalA, which formed crystals. These crystals were washed with hexanes and then recrystallized from 10% ethyl acetate in hexane, which yielded approximately 250 to 300 mg of white needles [28]. Moreover, twenty grams of dried *Salvia divinorum* leaves were macerated with 25 mL of a solution that contained 10% dimethyl sulfoxide and 0.1% 0.1 M hydrochloric acid. The solution was heated for approximately 3 h at 45 °C, then filtered, and the supernatant was stored at 4 °C. The concentration of SalA was determined by liquid chromatography–tandem mass spectrometry [29]. Finally, in another procedure, 600 g of leaves were pulverized and macerated with hexane (3.5 L, repeated three times). The residue was subsequently macerated with ethyl acetate (3.5 L, repeated three times). The final filtrates were concentrated at reduced pressure to remove the solvents, which yielded 16.7895 g of a dark green semi-solid mid-polar crude extract. It was determined that this concentrate contained 3.14 mg per gram of a mixture of salvinorins [30].

## 3. Pharmacodynamic Properties of Salvinorin A

SalA is a non-alkaloidal hallucinogen that acts as a potent and selective agonist of KORs [31,32]. More specifically, this compound has a high affinity for κ-1 but not κ-2 opioid receptors [33], and it lacks the capacity to modulate MOR. Therefore, this lack of interaction contributes to a lower risk of addiction potential and respiratory depression (Table 1) [34]. Unlike other hallucinogen compounds, SalA does not target 5-hydroxytryptamine receptor subtype 2A (5-HT_2A_R), which is the primary molecular target responsible for the effects of classical hallucinogens, such as psilocybin, mescaline, or lysergic acid diethylamide (LSD) [13,35,36,37]. 

In line with this, a placebo-controlled, randomized, double-blind study that involved the inhalation of 1 mg of vaporized SalA by 24 volunteers with prior experience in the use of psychedelics demonstrated that SalA diminished external sensory perception and elicited visual and auditory alterations. These effects were effectively prevented by naltrexone, a non-selective opioid receptor antagonist, but not by ketanserine, a 5-HT_2A_R antagonist [38]. Among the hallucinogenic effects of this compound having been described, a wide range of effects can be exhibited in a dose-dependent manner. Clinical data show evidence of disconnection from external reality, induced visions, auditory phenomena, and modified interoception after different doses of vaporized SalA. In this regard, lower doses produced somatic sensations, while higher doses resulted in a complete loss of contact with the body [39]. Furthermore, healthy volunteers exhibited increased plasma levels of prolactin and cortisol following the administration of vaporized SalA, providing evidence that salvinorin can influence the endocrine system. Interestingly, SalA has no effects like euphoria, cognitive deficits, or changes in vital signs [40].

Another pharmacodynamic property is that in vitro assays indicated that SalA can increase the dopamine transporter (DAT) activity, which, in turn, increases the clearance of dopamine in the striatum of rats [41]. Also, SalA extends its effects to influence another amine transporter as the serotonin transporter (SERT) produces a decrease. Meanwhile, norepinephrine transporter (NET) activity is not modulated [41]. Paradoxically, the effects of SalA are dose-dependent, suggesting a biphasic effect. Preclinical models showed an increase in dopamine levels in the nucleus accumbens (NAcc) after the systemic and chronic administration of small doses of SalA [42]. Similarly, low doses of SalA induced place preference and elevated dopamine levels in the NAcc of rats while decreasing dopamine levels in the caudate putamen; furthermore, the rewarding effects of SalA were prevented by the antagonism of KORs and cannabinoid receptors of type 1 (CB1Rs) [43]. These findings agree with other reports about the cross-talk regulation between KOR and the cannabinoid system induced by SalA [44,45], specifically an indirect activation of CB1R [46,47]. 

Furthermore, it has been reported that SalA exerted antinociceptive effects by participating in 5-hydroxytryptamine subtype receptor 1A (5-HT_1A_R), suggesting that this receptor is a pharmacological target of SalA [30]. Furthermore, SalA attenuated the inflammatory response by reducing the levels of tumor necrosis factor α (TNF-α) and interleukin 1β (IL-1β) after lipopolysaccharide treatment. It reduced the elevated levels of nitric oxide (NO) [48]. Furthermore, SalA might be able to induce vasodilation after the activation of nitric oxide synthase (NOS) and adenosine triphosphate (ATP)-sensitive potassium channels [49]. The extracts of *Salvia divinorum* decreased hyperalgesia induced by neuropathy and inflammation models, specifically through ligature of the sciatic nerve and carrageenan injection in the paws of rats [30,50]. 

The SalA mechanism of action results in the regulation of several neurologic targets. Some of the proteins involved in signaling pathways, such as extracellular signal-regulated protein kinase (ERK), can be modulated after the administration of SalA in a hypoxia/ischemia model in piglets [51]. ERK is a protein kinase protein implicated in regulating the metabolism, migration, survival, growth, proliferation, and differentiation of cells, which capture the surface receptor signals and then translocate to the nucleus [52]. Preclinical experiments suggested that participation in the phosphoinositide 3-kinase/protein kinase B/cyclic guanosine monophosphate (PI3K/AKT/cGMP) pathway is responsible for the neuroprotective effects of SalA on forebrain ischemia in rats [53]. 

## 4. Pharmacokinetic Profile of Salvinorin A

Several studies provided evidence to delineate the effects of SalA after this compound is administered in different ways and doses. The absorption of SalA occurs through the oral mucosa or the respiratory tract. When this compound is swallowed, it is rapidly hydrolyzed in the gastrointestinal system into its major inactive metabolite, Salvinorin B. SalA is quickly distributed, followed by accumulating in the brain, and then is rapidly eliminated. Its pharmacokinetic parameters are well correlated with its short psychoactive and physiological effects [54,55].

All the studies described in the literature indicate rapid action and short elimination half-lives for SalA, highlighting the role of carboxylesterase as the main enzyme involved in the hydrolysis of SalA in rat plasma [56]. Additionally, the absence of a protonated amine group, common to all previously known opioids, contributes to rapid metabolism, fast elimination, and a swift loss of activity [57]. 

In some instances, SalA presented low bioavailability and a lack of physiological or subjective effects when administrated in a sublingual way [58]. In contrast, when its compound was absorbed through the oral mucosa of humans, the initial effects were typically experienced within 5 to 10 min. The intensity of these effects increased rapidly over a few min, where it reached a plateau that lasted approximately 1 h. This temporal profile of effects was like oral ingestion. However, the vaporization or inhalation of SalA produced a quick experience of full effects in 30 s. The authors concluded that the oral mucosa appeared to function as a time-release buffer that gradually diffused SalA into the bloodstream [59]. Another report included subjects with histories of hallucinogen use. The inhalation of SalA (0.375–21 μg/kg) reached the peak of its effects after 2 min and rapidly dissipated. In addition, SalA produced dose-related dissociative effects and impairments in recall/recognition memory [60]. In a parallel pattern, other works described a similar effect that reached 2 min and then did not last more than 20 min after the inhalation of SalA [61]. 

On the other hand, pharmacokinetic parameters of animal models showed that after the intraperitoneal administration of SalA (10 mg/kg), the half-life time (t_1/2_) was 75 min, with a clearance (Cl/F) rate of 26 L/h/kg and a volume of distribution of 47.1 L/kg. In contrast, the brain t_1/2_ was shorter (36 min) than the plasma t_1/2_. Also, in this study, an in vitro assay demonstrated that SalA was a substrate for P-glycoprotein, UDP-glucoronosyl transferase (UGT)2B7, cytochrome P (CYP) D6, CYP1A1, CYP2E1, and CYP2C18 enzymes [62]. Then, p-glycoprotein was modulated by SalA, and this transport was involved in crossing the blood–brain barrier for many drugs [31]. 

Furthermore, non-human primates were used to study the SalA distribution in the brain and its physiological effects. Female baboons’ brains were studied by positron emission tomography, indicating an extremely rapid brain uptake that reached a peak of 3.3% of the administrated dose within 40 s, with a t_1/2_ of 8 min. SalA was distributed throughout the brain, with the highest concentration observed in the cerebellum and significant concentrations in the visual cortex. It was determined that SalA was excreted through the renal and biliary systems. Moreover, data suggest a direct relationship between the concentration of SalA and its physiological effects [28]. 

The sex differences in the pharmacokinetics of SalA were studied in primates, such as *Rhesus monkeys* (*n* = 4, 2 male and 2 female). The intravenous injection of SalA in the male monkeys showed that the elimination was rapid (t_1/2_ = 37.9 ± 5.6 min), with an area under the curve (AUC) = 572 ± 133 ng min/mL, whereas female monkeys showed a t_1/2_ = 80 ± 13.1 min, with an AUC = 1087 ± 46 ng min/mL [63]. Interestingly, the SalA treatment was significantly more potent in the females (50% effective dose (ED_50_) = 0.99 mg/kg) than in the male rats (ED_50_ = 3.94 mg/kg) submitted to paclitaxel-induced neuropathic pain [64]. Together, gender differences in the pharmacokinetics of SalA could partly explain these effects.

## 5. Toxicity and Safety Profile of Salvinorin A

The ethnobotanical uses of *Salvia divinorum* and its primary active component SalA were the subject of various studies regarding its potential toxicity and safety.

Despite SalA producing toxicity when using cellular viability assays, such as rat dopaminergic neural cells (N 27), human alveolar adenocarcinoma (A549) cells, human colorectal adenocarcinoma (Caco-2) cells, human hepatocellular carcinoma (Hep G2) cells, African green monkey kidney fibroblast cells (COS-7), and human embryonic kidney (Hek 293) cells [29], no intensive adverse effects have been reported in humans. The prevalent consumption of this drug among young people, along with the pharmacologic interest in treating many conditions, underscores the evident need to perform toxicity and safety studies. After the acute consumption of 200 to 500 µg of SalA in a vaporized form, this compound produces the same subjective response as the whole plant [59]. Psychotropic effects, blocking external sensory perception, modifying interoception and body awareness, dissociating effects, auditory modifications, memory impairment, and mystical perceptions are some of the most commonly experienced effects after consumption. Hallucinogenic effects were observed after 200 µg of SalA [39,59,60,61]. After consuming 1 mg of this compound, the typical hallucinogen effects are observed. Beyond the typical hallucinogenic effects of SalA, patients experience alterations in systolic blood pressure and cortisol levels [38].

Additionally, a double-blind, placebo-controlled, randomized study analyzed the subjective experiences of SalA in a chronic scheme consumption. Participants smoked either 1017 or 100 μg doses, administered two weeks apart in a counterbalanced order. After 8 weeks of follow-up, no variations in heart rate or blood pressure were reported; furthermore, 20 of the 23 participants reported aftereffects like becoming reflective and more emotionally sensitive, positive aftereffects (i.e., calm, relaxed, peaceful, happy), headache, fatigue, difficulty concentrating, more intuition, feelings of floating, and being more aware of beauty [65]. In support of this, in MacLean’s study on inhaled SalA, no participants reported adverse effects after one month post-administration [60]. Johnson and coworkers mentioned that volunteers experienced intense sensations and unusual, sometimes recurring, themes across sessions, such as revisiting childhood memories, cartoon-like imagery, and contact with entities [61]. 

Moreover, preclinical data indicate that medium polar extracts of *Salvia divinorum* produced sedative-like depressant properties, which altered the physiological sleep architecture in rats [66]. Furthermore, anxiolytic, depressive, and antidepressant effects are induced by SalA. However, these differences are due to the doses, concluding that low doses of this compound can induce antidepressant effects, while high doses produce depression and impair locomotor activity [67,68]. Finally, there is no existing evidence regarding toxicological assays in organs; however, this may be considered for future testing.

## 6. Salvinorin A and the Development of Synthetic Analogs

Even though the clinical application of SalA is limited by rapid metabolism, poor bioavailability, and psychoactive effects, it is important to develop synthetic analogs to enhance their therapeutic potential. As a KOR agonist, this unique property allows for potent KOR-mediated analgesia without engaging the MOR pathways typically associated with addiction and respiratory depression [54]. However, the short-term anti-pain effects elicited by SalA require frequent dosage or advanced delivery systems to maintain therapeutic levels. Although there is generally poor bioavailability, SalA demonstrates especially low oral bioavailability, affecting the efficiency of systemic delivery. Therapeutical high doses of SalA can induce dysphoria and other psychoactive effects, limiting its use in clinical studies [69].

In the last few years, hundreds of structural chain modifications to SalA were performed to improve its pharmacokinetic and pharmacodynamic properties. Accordingly, the C-2, C-6, and C-12 (the furan ring of SalA) positions of SalA (Figure 1) can offer binding stability with the KOR site. Herkinoin, herkamide, kurkinorin, salvidolin, methoxymethyl, the ethosymethyl ether of Salvinorin B, methyl salvinorin B 2-O-malonate, 2-O-cinnamoylsalvinorin B, and the β-Tetrahydropyranyl Ether of Salvinorin B are some examples of analogs of SalA that belong to the C-2 modifications, which exhibited different selectivities for KOR and MOR targets [70,71,72,73,74,75,76,77,78,79,80,81]. In line with this, the C-2 SalA analogs are the most advanced compounds, which were demonstrated to attenuate the nociceptive and inflammatory pain behaviors in several experimental preclinical models [50,71,82,83,84]. In this context, the SalA analog β-tetrahydropyran Salvinorin B showed anti-neuropathic, anti-inflammatory, and anti-nociceptive effects in a range of experimental murine models, where these effects were more potent compared with the SalA effects. The authors concluded that the C-2 modifications can alter the analgesic potency and efficacy of SalA, noting the risk of abuse [85]. Notwithstanding, not all synthetic analogs of SalA exhibit a better analgesic spectrum than SalA [86]. 

## 7. Physiopathology of the Trigeminal Sensory System and Molecular Mechanisms

**Figure 2:**
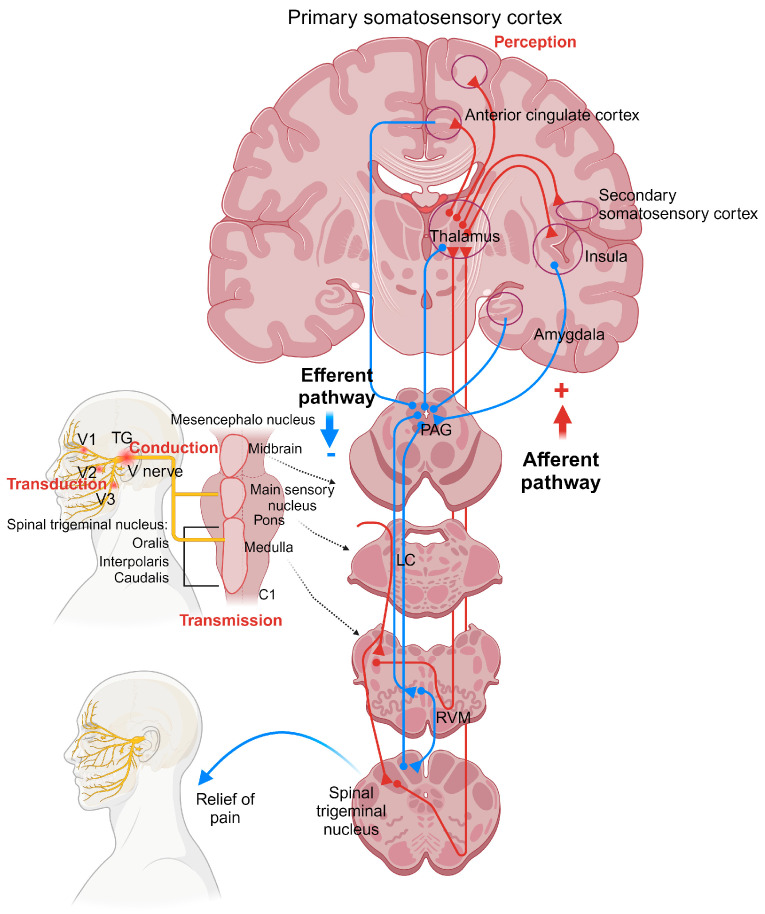
Pathophysiology of the trigeminal sensory system. Ascending (red circuit) and descending (blue circuit) pathways are involved in the control of pain processing in TN. The nociceptive signal is transduced into an electrical stimulus, which is conducted to the spinal nucleus, primarily the subnucleus caudalis. At this site, the peripheral neuron releases nociceptive neurotransmitters to the second-order neurons for transmission, and the signal then ascends through the corticotrigeminal projections to the thalamus. From there, the nociceptive message is transmitted to the insula, anterior cingulate cortex, and primary and secondary somatosensory cortices, where pain is perceived. In parallel with the pronociceptive pathways, a response from supraspinal structures, such as the insula, amygdala, thalamus, and anterior cingulate cortex, travels via the PAG-RVM pathway to the spinal trigeminal nucleus, producing antinociception. V nerve, trigeminal nerve; V1, ophthalmic nerve; V2, maxillary nerve; V3, mandibular nerve; TG, trigeminal ganglion; PAG, periaqueductal gray; RVM, rostroventromedial medulla; LC, locus coeruleus. Created in BioRender. Quiñonez-Bastidas, G. (2024) https://BioRender.com/u43z819. Accessed on 24 November 2024.

The trigeminal system transmits motor and sensory information from the craniofacial region; hence, a trigeminal nerve injury conduces chronic neuropathic pain syndrome, a common type of orofacial pain [87]. TN is produced by the activation of nociceptors at the ends of primary afferents fibers (Aδ or C nerve fibers) from the branches V1 (ophthalmic), V2 (maxillary), and V3 (mandibular), in which the nerve damage is transduced in an electrical neuronal input, followed by conduction along the fiber to the somas of trigeminal ganglia (TGs), where the nociceptive stimulus is increased by the synthesis of neurochemicals and gene transcription (Figure 2) [88,89]. The afferent neurons project at the V brainstem complex, which is subdivided into the main sensory nucleus and the spinal tract nucleus. Moreover, the tract nucleus is formed by the oralis, interpolaris, and caudalis. This last one is the most important site for the transmission of nociceptive input [87,90]. Consequently, the primary sensory fiber releases neurotransmitters to the second-order neuron at the nucleus caudalis laminate I, II, V, and VI; then, a nociceptive message is sent via ascending pathways to a third-order neurons in the brainstem structures, like the thalamus, insular cortex, and cerebral cortex to the perception of pain [91].

Trigeminal neuropathic pain can be exacerbated or modulated by distinct processes. The first process involves peripheral sensitization; the nerve injury releases inflammatory compounds, such as amines, neurotransmitters, and interleukins (i.e., 5-HT, substance P (SP), IL-1β, TNF-α) [91,92,93]. Also, in the TG neurons, a lesion of trigeminal nerve roots lead to a stress process with an increase in reactive oxygen species (ROS) production, which increases the activity of the transient receptor potential Ankyrin1 (TRPA1) channel and an influx of cations, like Na^+^ and Ca^2+^ [94]. Likewise, the neuroinflammation component plays a specific role in increasing the proinflammatory cytokines, like IL-1β, interleukin 6 (IL-6), and TNF-α [95,96,97]. In addition, genetic mutations can produce channelopathies (in Na^+^, K^+^, Ca^2+^, and C1^−^ channels) to cause neuronal hyperexcitability, and some specific mutations were identified in the γ-aminobutyric acid type A (GABA_A_) receptor, voltage-gated calcium channel (Cav) 3.2., and voltage-gated sodium channel (Nav) 1.3 and Nav 1.7 [98,99,100]. Moreover, the abnormal function of ion channels, like Na^+^, K^+^, Ca^2+^, Cl^−^, and transient receptor potential (TRP) channels produces an increase in axonal hyperexcitability, increasing the conduction of nociceptive transmitters [101].

In another process, central sensitization occurs to amplify the nociceptive information in neurons of the nucleus caudalis. The transient receptor potential vallinoid 1 (TRPV1) channel is increased in the central sensitization [102], which is also linked to the development of hyperalgesia. Due to the role of channelopathies in the trigeminal pain process, the first line of treatment for this condition is focused on the administration of gabapentinoids drugs, which decrease the neuronal excitability to produce analgesia [103].

Central sensitization is an important process involved in the maintenance of painful symptoms in patients with TN [104]. The aberrant process begins with the release of glutamate and other neurotransmitters to the second-order neuron. N-methyl-D-aspartic acid (NMDA) receptors in the second-order neurons are activated, which subsequently activates α-amino-3-hydroxy-5-methyl-4-isoxazolepropionic acid (AMPA) receptors to increase the intracellular influx of Ca^2+^. The increased levels of Ca^2+^ activate the protein kinase A (PKA), protein kinase C (PKC), and ERK1/2 proteins, which promote the phosphorylation of other receptors. In this way, an induction of NOS occurs, as well as the consequent production of NO. Furthermore, a reduction in K^+^ currents is induced, contributing to the feedback of the neuronal hyperexcitability and the up-activation of transcription factors genes, like c-Fos, neurokinin 1 (NK1), tropomyosin receptor kinase B (TrkB), and the cyclooxygenase 2 (Cox-2) enzyme. In parallel, γ-aminobutyric acid (GABA) release from inhibitory interneurons is decreased, producing disfunction in inhibitory interneurons, contributing to synaptic plasticity changes [105,106]. Preclinical and clinical studies showed evidence for how gabapentin and pregabalin, where both drugs are considered first-line treatments for the symptoms of neuropathy in TN, display their effectiveness through the blockade of the α2δ calcium channel subunit, which, in turn, decreases the neurotransmitters released to the second-order neurons at the spinal trigeminal nucleus caudalis, resulting in the suppression of central sensitization and the relief of pain [107,108]. Also, TRPV1 activation on afferent terminals promotes Ca^2+^ influx, which enhances the movement of intracellular glutamate vesicles and their subsequent release [109]. Furthermore, blocking the TRPV1 expressed on the central terminals of primary sensory neurons attenuates the central sensitization and neuropathic pain [110]. 

Furthermore, astrocytes and microglial cells, as well as purinergic (i.e., P2X4, P2Y12) and IL-1β receptors, are overexpressed on brainstem sections during the process of trigeminal sensitization, underlining the role of non-neuronal cells in the maintenance of central sensitization, which correlates with allodynic behaviors [111]. Consistent with this, the purinergic receptors P2X and P2Y are also expressed in neuronal cells. These receptors are activated by endogenous ATP, and then ATP is implicated in the development of central sensitization in nociceptive neurons at the trigeminal subnucleus caudalis [112].

Although there are ascending spinothalamic tracts that send pain messages to brainstem structures, there are also descending pathways from the periaqueductal gray (PAG) neurons to the locus coeruleus (LC) and rostral ventromedial medulla (RVM), specifically through the nucleus raphe magnus (NRM) to finally activate analgesia in the spinal trigeminal nucleus caudalis [113,114]. GABAergic projections from the PAG produce antinociception through the excitation of OFF cells in the RVM and inhibition of ON cells [115]. ON cells at the RVM are associated with the hyperalgesia process that facilitates the activity of the nocifensive reflex, meanwhile OFF cells inhibit the nociceptive transmission [116,117]. Also, norepinephrine and serotonin projections from the RVM to the spinal trigeminal subnucleus caudalis can modulate pain [93,118]. Regarding this circuit, opioidergic and cannabinergic projections activate the antinociceptive descending pathways [119,120]. The evidence indicates that stimulation of the primary somatosensory cortex inhibits nociceptive behavior in the spinal trigeminal subnucleus caudalis, suggesting the role of endogenous pain control through the corticotrigeminal descending pathway [121]. Furthermore, clinical data using functional magnetic resonance imaging in patients with TN show increased activity in the spinal trigeminal nucleus, thalamus, primary and secondary somatosensory cortices, anterior cingulate cortex, insula, premotor and motor cortex, prefrontal areas, putamen, hippocampus, and brainstem after patients received a painful stimulus in the trigger zone of TN [122]. Together, all evidence in TN indicates the impairment of descending control pain pathways, where the neuroplasticity of this circuit switches from PAG-RVM control to PAG-RVM, facilitating the pain process; thus, this provides one of the targets for the future pain control for this condition (Figure 2).

## 8. Experimental Models to Induce Trigeminal Pain

TN is a condition characterized by neuropathic pain with conspicuous paroxysmal and shock-like pain episodes. Given the multifactorial causes of TN, several experimental animal models tried to mimic the symptoms or features of this disorder [123]. All the experimental models aimed to translate the human symptoms of this condition to animal-behavior-evoked or -non-evoked pain to understand the pain pathways involved in the development and maintenance of TN, and to explore the molecular basis of the pathology involved in new drug design to treat the pain [124]. 

Clinically, spontaneous and evoked pain as allodynia and thermal hyperalgesia (cold and heat) are common features of pain in TN [125]. Likewise, experimental models of TN in rodents permit reproducing spontaneous nociception as facial expressions (orbital tightening, nose bulge, check bulge, ear position, and whisker change, which are evaluated by the Grimace scale) [126] or facial grooming [127]. Evoked nociception is observed in several models, such as mechanical allodynia (von Frey filaments) and thermal nociception (cold and heat stimuli) [128,129]. Some examples of the most common TN models in rodents are induced by chemical, surgical, and genetic modifications or a combination of genetic modification with surgery or chemical induction [130] (Table 2). Modeling the pathophysiology and clinical characteristics of trigeminal neuropathy in animal models remains a significant challenge. A thorough understanding of the underlying pathophysiology is crucial for developing novel pharmacological targets to alleviate the suffering of individuals affected by this condition. While experimental models provide valuable insights into the mechanisms of trigeminal neuropathy, they also possess inherent limitations [131]. Some models induce pain alongside depressive-like behaviors, making it essential to differentiate between these outcomes carefully. Additionally, the absence of complex vascular features in certain models, or their incomplete representation of pathological conditions, presents further drawbacks. Not all models can replicate the full spectrum of pain symptoms observed clinically. In this condition, the pain is sharp, electric-shock-like, stabbing, or lancinating pain. Furthermore, pain assessments in these models require animal restriction or conditioning [123,129,132]. These limitations are critical for developing new therapeutic approaches and are essential for ensuring the effective translation of scientific findings from preclinical to clinical models.

## 9. Cross-Talk Between Salvinorin A and Trigeminal Pain Regulation

Given the role of KORs in the antinociceptive process, SalA demonstrated its effectiveness in spinal-mediated pain (Table 3) [145]. Moreover, pharmacodynamic studies indicated that antinociceptive effects induced by SalA not only involve KOR but also CB1R and 5-HT_1A_R pathways [15,146]. However, the antinociceptive effect of this psychotropic drug and its molecular targets on the trigeminal-mediated pain process remains unproven. In this regard, the endogenous κ-opioid system is impaired after trigeminal damage. Moreover, animals develop mechanical allodynia and spontaneous pain-like behavior. Both allodynia and spontaneous pain are increased in mice with KOR gene deletion compared with mice with the KOR system. Furthermore, the intraperitoneal injection of the KOR agonist U50 488 reversed the allodynia in wild-type mice but not in KOR-knockout mice [147]. KOR endogenous systems are important to develop pain and recovery in animals. Notwithstanding, a recent report indicates that blocking the KOR system reduces mechanical hyperalgesia and anxiety-like behavior in a model of CCI-IoN in rats. The systemic administration of nor-binaltorphimine, as well as in the site of TG and central amygdala (CeA), produced a decrease in pain behavior; in contrast, administration in the spinal trigeminal nucleus caudalis did not show an antiallodynic effect [148]. In line with this, the antinociceptive effect of the κ-opioid system could involve supraspinal regulation of emotions like anxiety. For instance, the blocking of KOR in the CeA decreases mechanical hyperalgesia and affective and anxiety-like behavior in a model of functional pain syndromes induced in stress situations [149]. Moreover, KORs play a role in chronic orofacial pain, where a recent study demonstrated that female rats exhibited a loss of descending pain inhibitory pathways. This phenomenon did not occur in males, while in females, it was prevented by KOR antagonists, suggesting that KORs play a pronociceptive role at the central level [150]. In contrast, recent findings indicate that peripheral activation of KORs produces potent analgesia with fewer side effects [36,151]. Although there are few studies on the analgesic effects and role of KORs in the trigeminal system, some authors suggest that the analgesic mechanisms of KORs involved in orofacial pain may be due to the peripheral activation of these receptors [152]. Additionally, it was observed that KORs are capable of activating cannabinoid receptors peripherally to induce analgesia [37]. Taken together, the role of the KORs on TN depends on the site of action, the dose, and the regulation of emotions in the amygdala.

**Figure 3:**
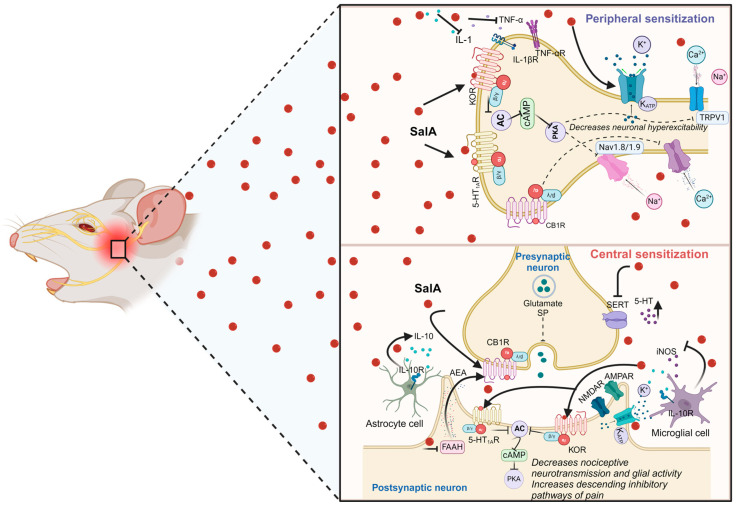
The role of SalA in trigeminal neuralgia: A plausible explanation for its potential activation targets involves three primary pathways—KORs, CB1Rs, and 5-HT_1A_Rs. The upper panel depicts the mechanisms of peripheral sensitization, while the lower panel illustrates central sensitization and highlights the prospective targets of SalA. The mechanisms of SalA are intrinsically linked to the cannabinoid system, where it activates CB1Rs, leading to the inhibition of presynaptic transmission and a subsequent reduction in the release of nociceptive neurotransmitters, such as glutamate and SP. This activation also inhibits the Ca^2+^ channel activity. The decrease in glutamate release attenuates the activation of NMDAR and AMPAR channels, thereby reducing central sensitization. Additionally, SalA inhibits the FAAH enzyme, resulting in elevated AEA levels, which further activate presynaptic CB1R, contributing to analgesic effects. Moreover, SalA engages the serotonergic system by specifically activating 5-HT_1A_Rs, which inhibit the AC enzyme activity and reduces the cAMP levels and PKA activity, thereby inducing analgesia. SalA also inhibits SERT, preventing 5-HT reuptake and increasing its extra synaptic levels. In addition, SalA acts on KORs, inhibiting the AC-cAMP-PKA pathway, which subsequently blocks TRPV1 and Nav1.8/1.9 channels, reducing neuronal hyperexcitability. SalA also exerts anti-inflammatory effects by inhibiting the release of pro-inflammatory cytokines, such as IL-1 and TNF-α, thereby reducing peripheral sensitization. In microglial cells, SalA suppresses iNOS enzyme activity, mitigating central sensitization. Furthermore, SalA enhances the release of IL-10 from astrocytes, which activates IL-10Rs in the glial cells, regulating glial inflammatory products and further reducing central sensitization while promoting analgesia. Arrows show the mechanisms by which SalA induces antinociception. At the same time, inhibitory lines indicate how SalA suppresses proteins, enzymes, and transporters, and reduces the release of cytokines and enzymes from glial cells, all of which are involved in pronociceptive processes. Dashed lines represent the indirect inhibitory action of SalA on ion channels and neurotransmitters. AC, adenylyl cyclase; AEA, arachidonoyl ethanolamine; AMPAR, α-amino-3-hydroxy-5-methyl-4-isoxazolepropionic acid receptor; cAMP, cyclic adenosine monophosphate; CB1R, cannabinoid receptor type 1; 5-HT, 5-hydroxytryptamine; 5-HT_1A_R; 5-hydroxytryptamine receptor 1A; IL-1, interleukin 1; IL-1R, interleukin 1 receptor; IL-10, interleukin 10; IL-10R, interleukin 10 receptor; iNOS, inducible nitric oxide synthase; K_ATP_, ATP-sensitive potassium channel, KOR, κ-opioid receptor; TNF-α, tumor necrosis factor α; TNF-αR, tumor necrosis factor α receptor; Nav, voltage-gated sodium channels; NMDAR, N-methyl-D-aspartic acid receptor; PKA, protein kinase A; SERT, serotonin transporter; TNF-α, tumor necrosis factor α; TNF-αR, tumor necrosis factor α receptor; TRPV1, transient receptor potential vallinoid 1; SP, substance P. Created in BioRender. Quiñonez-Bastidas, G. (2024) https://BioRender.com/u43z819. Accessed on 24 November 2024.

Furthermore, it is well known that trigeminal pain regulation encompasses peripheral and central sensitization (Figure 3). The nerve injury produces a peripheral sensitization characterized by the accumulation of macrophages and satellite cells in the site of TG, which, in turn, release neurotransmitters and proprioceptive molecules enhancing the neuron excitability and activating the second-order neurons in trigeminal spinal subnucleus caudalis and upper cervical spinal cord (C1/C2), which produce an aberrant circuit that increases the central sensitization [141,147,156]. Parallelly, the neuroinflammation produced after the trigeminal nerve damage impairs the normal function of serotonergic projections from the RVM to the spinal trigeminal subnucleus caudalis and C1/C2 regions [157,158]. On the other hand, the participation of ascending and descending pathways was described after damage to the orofacial region. The RVM has a crucial role in the modulation of pain; for instance, the administration of the 5HT_1A_ agonist into the RVM produces analgesia [159,160,161]. In line with this, SalA exhibited anti-inflammatory and antinociceptive effects through activating the 5-HT_1A_Rs [15]. Moreover, the activation of this receptor displayed a strong antiallodynic effect on experimental trigeminal pain in rats [162,163]. In addition, sumatriptan, an agonist of the 5-HT_1A/1B/1D_Rs, produced analgesia in patients with trigeminal pain [164].

Interestingly, the activation of CB1Rs was described to explain the antinociceptive, anti-inflammatory, and anti-neuropathic effects of SalA (Figure 3). Cannabinoid pathways were activated after the systemic and insular cortex administration of SalA, which suggests that their effects involved peripheral, central, and supraspinal anti-pain circuits [17,146]. Therefore, CB1Rs are critical in peripheral input inhibition and trigeminal pain processes; likewise, these are expressed in TG large-size myelinated neurons and widely distributed in the brain, which offers the possibility to activate supraspinal antinociceptive pathways [165,166]. Moreover, the activation of KORs and CB1Rs mediated by SalA were responsible for producing antidepressant- and anxiolytic-like behaviors in rodents, where both mood conditions are linked to reducing several pain conditions in humans. In addition, SalA can modify the cannabinoid endogenous system by reducing fatty acid amide hydrolase (FAAH) activity in the amygdala [67]. FAAH is an enzyme that acts by degrading the endogenous cannabinoid anandamide to oleic and arachidonic acid. Hence, the inhibition of this produces an increase in anandamide that is able to bind to CB1R, cannabinoid receptor type 2 (CB2R), and TRPV1, which are all involved in antinociceptive pathways [167]. Accordingly, scientific reports highlight the potential therapeutic benefits of FAAH inhibition to produce analgesic, anti-depressive, and anxiolytic effects [168,169,170]. The inhibition of FAAH prevented the development of allodynia. Likewise, it induced changes in calcitonin gene-related peptide (CGRP) and cytokines in the medulla, cervical spinal cord, and TG in experimental TN [171,172]. Concomitantly, anandamide modulated the central sensitization process in the trigeminal system at the cervical spinal segment C1/C2 [173]. In line with this, several models of trigeminal sensory nerve injury showed that trigeminal central sensitization is responsible for the development of pain hypersensitivity, noting the role of astrocytes and microglia in the feedback of nociceptive process [174,175,176]. Moreover, the roles of IL-1β, TNF-α, and other cytokines are important in the activation of glial cells at the spinal trigeminal nucleus after nerve injury [177]. Parallelly, a significant reduction in mechanical facial pain induced by the CCI-IoN model in rats was observed when IL-1β and TNF-α release decreased in TG neurons [178]. Hence, SalA may play a crucial role in the attenuation of peripheral and central sensitization phenomena by inhibiting the adenylyl cyclase (AC) enzyme and its second messenger cAMP, which, in turn, inhibits the protein kinase A (PKA) in sensory primary neurons. Furthermore, SalA regulates iNOS and IL-10 in astrocytes and microglial cells in the spinal cord. Both processes modulate and are responsible for nociceptive behaviors induced by damage to the trigeminal nerve [48,153,154].

Finally, SalA can activate the ATP-sensitive potassium (K_ATP_) channels located on the peripheral terminal of trigeminal ganglion neurons and have essential functions in neuronal hyperpolarization, which decreases neuron excitability, and thus, induces analgesia [49,179]. Scientific evidence suggests that SalA is able to modulate the KORs, 5-HT_1A_Rs, and endogenous cannabinoid system, including the activation of the CB1Rs and FAAH enzyme regulation, as well as extending its mechanism of action through the indirect activation of other receptors, ion channels, and the role of glial cells on trigeminal central sensitization, which might be an emergent therapeutic approach to relieve TN (Figure 3).

## 10. Concluding Remarks and Future Perspectives for Salvinorin A in the Treatment of Trigeminal Neuralgia

The extensive scientific reports on the antinociceptive, anti-inflammatory, and anti-neuropathic effects of SalA, as well as the identification of pharmacological targets in non-painful experimental models, highlight the importance of the role of SalA in the attenuation of TN as one of the hot points of research. However, the potential attenuation of pain-related behaviors induced by TN through the action of SalA remains a hypothesis. Due to its properties, SalA is considered a promising drug for treating this painful condition, in which the KOR, CB1R, and 5-HT_1A_R systems play key roles in the modulation of the trigeminal sensory complex. SalA, a non-addictive compound, is considered a promising therapeutic agent in developing therapies for managing TN. It is therefore recommended that preclinical studies be conducted that assess not only its analgesic potential but also the underlying mechanisms of action and its neuropharmacological and toxicological profile. Finally, future experimental designs should focus on (1) optimizing the SalA dose to mitigate the central nervous system depressant effect and hallucinations associated with using SalA and (2) developing synthetic analogs that address the SalA pharmacokinetic limitations while enhancing its analgesic properties. These approaches position SalA as a promising treatment for TN, offering significant advantages in terms of toxicity, tolerability, and safety compared with currently marketed anti-pain drugs.

## Figures and Tables

**Table 1:** Main side effects associated with opioid-induced analgesia.

Opioid Receptor Subtype	Side Effect
MOR	Nausea, hyperalgesia, tolerance, dependence, addiction, constipation, sedation, and respiratory depression
KOR	Sedation, dysphoria, and hallucinations
DOR	Constipation and convulsive effects

MOR, μ-opioid receptor; KOR, κ-opioid receptor, DOR, δ-opioid receptor.

**Table 2:** Animal models of trigeminal neuralgia and their spontaneous and evoked nociceptive behaviors.

	Animal Model of TN	Species	Nociceptive Behavior
Chemical induction	Formalin injection [133,134].	Rats and mice	Face grooming
	CFA injection [127,135].	Rats and mice	Face grooming Mechanical hyperalgesia
	Cobra venom injection in infraorbital nerve (IoN) trunk [136].	Rats	Mechanical allodynia
Surgery	Chronic compression of the trigeminal (CCT) nerve root [137,137].	Rats	Thermal hyperalgesia (heat) Mechanical allodynia Face grooming
	Trigeminal inflammatory compression (TIC) of the infraorbital nerve [138].	Mice	Mechanical allodynia Cold allodynia
	Chronic constriction injury of the distal infraorbital nerve (dIoN-CCI) [139,140].	Rats and mice	Face grooming Mechanical allodynia
	Partial infraorbital nerve ligation (pIoNL) [141,142].	Rats and mice	Face grooming Mechanical allodynia
	Infraorbital nerve chronic constriction injury CCI-IoN [143].	Rats and mice	Mechanical allodynia Face grooming Thermal hyperalgesia (heat) Grimace scale
Genetically modified	GABRG1 p.Cys188Trp mutation (GABA_A_ receptor Cl^−^ channel) [99].	Mice	Mechanical allodynia Pain-like behavior
	TRESK knockout (TWIK-related spinal cord K^+^ channel) [144].	Mice	Cold allodynia Thermal hyperalgesia (heat) Mechanical allodynia

**Table 3:** SalA effect in experimental pain-like behaviors induced by inflammatory, nociceptive, and neuropathic models.

Author and Year	Experimental Model of Pain	Animal Species	Dose/Route of SalA	Salvinorin Effect on Pain Like Behaviors
Paton et al., 2022 [64]	Paclitaxel-induced neuropathic pain	Male and female C57BL/6J mice	ED_50_ 0.99 and 3.94 mg/kg s.c. for mechanical and 0.60 and 1.03 mg/kg s.c. for cold allodynia in females and males, respectively. Cumulative effects, followed by administration every 30 min.	SalA cumulative effect decreased the mechanical and cold allodynia in neuropathic mice. SalA effects were more potent in female than male mice, and SalA treatment was more potent than morphine treatment in female mice. SalA effect on cold allodynia was not different between the sexes, but the antiallodynic effect was more potent than the morphine treatment.
Paton et al., 2020 [50]	Hot plate test Formalin test	Male B6-SJ mice	2 mg/kg i.p. 2 mg/kg i.pl.	SalA increased the paw withdrawal latency in the hot plate test and decreased the pain score in both phases of the formalin test.
Coffeen et al., 2018 [17]	Sciatic nerve ligature (SNL) model	Wistar rats	11.55 nM/µL at 2 µL/min i.c.	SalA increased the paw withdrawal latency to thermal and mechanical stimulus, which was interpreted as an anti-allodynic effect, through the participation of KORs and CB1Rs.
Jamshidi et al., 2015 [153]	Prostaglandin E2 (PGE_2_)-induced thermal allodynia	Male Sprague Dawley rats	0.1 µg i.pl.	SalA increased the paw withdrawal latency in the radiant heat stimulus, which was interpreted as an anti-allodynic effect; then, it was able to increase the activity of c-Jun N-terminal kinase (JNK) and inhibit cyclic adenosine monophosphate (cAMP) in primary sensory neuron cultures.
Paton et al., 2017 [85]	Paclitaxel-induced neuropathic pain Formalin-induced inflammatory pain Hot water tail-withdrawal test	Male mice B6-SJ	EC_50_ 3.3 and 0.59 mg/kg i.p. (mechanical and cold allodynia test, respectively) 2 and 2.1 mg/kg i.p. for inflammatory and nociceptive test.	SalA reduced the cold and mechanic allodynia, and then produced an anti-inflammatory and antinociceptive effect.
Guida et al., 2012 [154]	Long-lasting formalin-induced allodynia	C57BL/6J mice	2.0 mg/kg i.p. daily for 7 days	SalA significantly reduced mechanical allodynia and spinal neuronal hyperexcitability. Then, the antiallodynic effect involved KORs and CB1Rs; likewise, modulation of inducible nitric oxide synthase (iNOS) and interleukin 10 (IL-10) in astrocytes and microglial cells in the spinal cord.
Fichna et al., 2012 [146]	Experimental colitis model induced by intracolonic instillation of mustard oil	C57BL/6J mice	3 mg/kg i.p. and 10 mg/kg intracolonic	SalA exhibited significant anti-inflammatory and antinociceptive effects in the pain-related behaviors (licking, stretching, squashing, and abdominal retractions) through the activation of KORs and CB1Rs.
Walentiny et al., 2010 [47]	Tail flick test	Male ICR mice	1–3 mg/kg i.p.	SalA produced dose-dependent antinociceptive effects, which were mediated by KORs.
McCurdy et al., 2006 [145]	Tail flick test Hot plate test Acetic acid abdominal constriction assay	Male Swiss mice	0.5, 1.0, 2.0, and 4.0 mg/kg	SalA increased the latency in the tail flick and hot plate test, which was interpreted as an antinociceptive effect, and reduced abdominal constrictions. The antinociceptive effects of SalA were mediated by KOR pathway activation.
John et al., 2006 [155]	Tail flick test	Male CD-1 mice	13.9–23.1 nmol i.t.	SalA exhibited a significant antinociceptive effect in the formalin-induced pain model, which reduced the pain responses in mice.
Ansonoff et al., 2006 [33]	Tail flick test	Male mice C57BL6/J 129S6 and knock out KOR 1	1.5–15 µg i.c.v.	SalA increased the percentage of maximum possible antinociceptive effect in mice. Antinociception was mediated through KOR 1 but not by KOR 2.

cAMP, cyclic adenosine monophosphate; CB1R, cannabinoid receptor type 1; EC_50_, half-maximal effective concentration; ED_50_, 50% effective dose; i.c., insular cortex; i.c.v., intracerebroventricular; IL-10, interleukin 10; iNOS, inducible nitric oxide synthase; i.p., intraperitoneal; i.pl., intraplantar; i.t., intrathecal; i.v., intravenous; JNK, c-Jun N-terminal kinase; KOR, κ-opioid receptor; PGE_2_, prostaglandin E2.

## Abbreviations

A549, human alveolar adenocarcinoma cells; AC, adenyl cyclase; AEA, arachidonoyl ethanolamine; AKT, protein kinase B; AMPA, Alpha-amino-3-hydroxy-5-methyl-4-isoxazolepropionic-acid; AMPAR, Alpha-amino-3-hydroxy-5-methyl-4-isoxazolepropionic-acid receptor; ATP, adenosine triphosphate; AUC, area under the curve; Caco-2, human colorectal adenocarcinoma cells; cAMP, cyclic adenosine monophosphate; Cav; voltage-gated calcium channel; CB1R, cannabinoid receptor type 1; CB2R, cannabinoid receptor type 2; CCI-IoN, infraorbital nerve chronic constriction injury; CCT, chronic compression of the trigeminal; CeA, central amygdala; CFA, complete Freund’s adjuvant; cGMP, cyclic guanosine monophosphate; CGRP, calcitonin gene-related peptide; Cl/F, clearance; COS-7, African green monkey kidney fibroblast cells; COX-2, cyclooxygenase 2; CYP, cytochrome P; DAT, dopamine transporter; dIoN-CCI, chronic constriction injury of the distal infraorbital nerve; EC_50_, half-maximal effective concentration; ED_50_, 50% effective dose; ERK, extracellular signal-regulated protein kinase; FAAH, fatty acid amide hydrolase; GABA, γ-aminobutyric acid; GABA_A_, γ-aminobutyric acid type A receptor; Hek 293, human embryonic kidney cells; Hep G2, human hepatocellular carcinoma cells; 5-HT, 5-hydroxytryptamine; 5-HT_1A_R, 5-hydroxytryptamine type 1A receptor; i.c., insular cortex; i.c.v., intracerebroventricular; IL-1β, interleukin 1β; IL-1βR, interleukin 1β receptor; IL-6, interleukin 6; IL-10, interleukin 10; iNOS, inducible nitric oxide synthase; IoN, infraorbital nerve; i.p., intraperitoneal; i.pl., intraplantar; i.t., intrathecal; JNK, c-Jun N-terminal kinase; K_ATP_, ATP-sensitive potassium channel; KOR, κ-opioid receptor; LC, locus coeruleus; LSD, lysergic acid diethylamide; MOR, µ-opioid receptors; N 27, rat dopaminergic neural cells; NAacc, nucleus accumbens; Nav, voltage-gated sodium channel; NET, norepinephrine transporter; NK1, neurokinin 1; NMDA, N-methyl-D-aspartic acid; NMDAR, N-methyl-D-aspartic acid receptor; NO, nitric oxide; NOS, nitric oxide synthase; NRM, nucleus raphe magnus; PAG, periaqueductal gray; PD, pharmacodynamic; PGE, prostaglandin E; PI3K, phosphoinositide 3-kinase; PK, pharmacokinetic; PKA, protein kinase A; PKC, protein kinase C; PIoNL, partial infraorbital nerve ligation; ROS, reactive oxygen species; RVM, rostral ventromedial medulla; SalA, salvinorin; SERT, serotonin transporter; SNL, sciatic nerve ligature; SP, substance p; t_1/2_: half-life time; TG, trigeminal ganglion; TIC, trigeminal inflammatory compression; TN, trigeminal neuralgia; TNFα, tumor necrosis factor α; TNF-αR, tumor necrosis factor α receptor; TRESK, TWIK-related spinal cord K^+^ channel; TrkB, tropomyosin receptor kinase B; TRP, transient potential receptor; TRPA1, transient receptor potential ankyrin 1; TRPV1, transient receptor potential vallinoid 1; UGT, UDP-glucoronosyl transferase.
